# Ubiquitin–proteasome system, lipid metabolism and DNA damage repair are triggered by antipsychotic medication in human oligodendrocytes: implications in schizophrenia

**DOI:** 10.1038/s41598-020-69543-5

**Published:** 2020-07-28

**Authors:** Gabriela Seabra, Valéria de Almeida, Guilherme Reis-de-Oliveira, Fernanda Crunfli, André Saraiva Leão Marcelo Antunes, Daniel Martins-de-Souza

**Affiliations:** 1 Laboratory of Neuroproteomics, Department of Biochemistry and Tissue Biology, Institute of Biology, University of Campinas (UNICAMP), Rua Monteiro Lobato, 255, Campinas, SP 13083-862 Brazil; 2 Experimental Medicine Research Cluster (EMRC), University of Campinas, Campinas, SP Brazil; 3 D’Or Institute for Research and Education (IDOR), São Paulo, Brazil

**Keywords:** Biochemistry, Molecular biology, Neuroscience, Systems biology

## Abstract

Schizophrenia is a chronic, severe and disabling psychiatric disorder, whose treatment is based on psychosocial interventions and the use of antipsychotic drugs. While the effects of these drugs are well elucidated in neuronal cells, they are still not so clear in oligodendrocytes, which play a vital role in schizophrenia. Thus, we aimed to characterize biochemical profiles by proteomic analyses of human oligodendrocytes (MO3.13) which were matured using a protocol we developed and treated with either haloperidol (a typical antipsychotic), clozapine (an atypical antipsychotic) or a clozapine + d-serine co-treatment, which has emerged lately as an alternative type of treatment. This was accomplished by employing shotgun proteomics, using nanoESI-LC–MS/MS label-free quantitation. Proteomic analysis revealed biochemical pathways commonly affected by all tested antipsychotics were mainly associated to ubiquitination, proteasome degradation, lipid metabolism and DNA damage repair. Clozapine and haloperidol treatments also affected proteins involved with the actin cytoskeleton and with EIF2 signaling. In turn, metabolic processes, especially the metabolism of nitrogenous compounds, were a predominant target of modulation of clozapine + d-serine treatment. In this context, we seek to contribute to the understanding of the biochemical and molecular mechanisms involved in the action of antipsychotics on oligodendrocytes, along with their possible implications in schizophrenia.

## Introduction

Schizophrenia is a severe, chronic, and disabling psychiatric disorder characterized by behavioral disturbances, abnormal mental functions and a heterogeneous combination of symptoms^1,2^, being one of the most important public health problems worldwide^1^. The symptoms are categorized as positive, negative and cognitive^1,2^, that usually manifest around the age of 16–30 years^3^. Treatment is based on a combination of antipsychotic drugs and psychosocial interventions^1,2^. Antipsychotics are divided into typical (first-generation) and atypical (second-generation) drugs^1^. A common pharmacological property between them is that they both block the dopamine D2 receptor^1^.

First-generation antipsychotics, such as chlorpromazine and haloperidol, are effective in reducing positive symptoms; however, they are minimally efficient for negative and cognitive symptoms^4,5^. Second-generation antipsychotics, such as clozapine, olanzapine, quetiapine, and risperidone, in turn, are more effective in reducing negative symptoms and improving global cognition than typical drugs. Moreover, they are associated with a lower occurrence of extrapyramidal effects and lower rates of treatment discontinuation and long-term relapse^1,4,5^. However, they also have side effects, such as weight gain and sedation^4–6^, and are associated with a higher cardiometabolic risk when compared to typical antipsychotics^1^. For this study, haloperidol was selected as typical antipsychotic, since it has been widely used in the clinic to treat the positive symptoms^7, 8^. As other typical drugs, haloperidol treatment is associated with extrapyramidal side effects^7,9^. Clozapine, the selected atypical drug, has a limited clinic use due the agranulocytosis. However, its efficacy is superior to many antipsychotic drugs, both typical and atypical^10^. Clozapine can also reduce suicide risk and is indicated in refractory schizophrenia treatment^11^.

The administration of D-serine with antipsychotics has been shown to be more effective in relieving some symptoms of schizophrenia, especially negative symptoms, when compared to the administration of antipsychotics alone^11^. D-serine is an endogenous N-methyl-D-aspartate (NMDA) receptor co-agonist that plays an important modulatory role in binding to the receptor’s glycine sites (NR1/NR2 subunits)^12^. It is for this reason that this amino acid has been tested as a potential therapeutic agent for schizophrenia in combination with atypical antipsychotics^13^.

A widely accepted hypothesis for the origin of schizophrenia is that variations in several risk genes interact with environmental stimuli, affecting brain development and function, leading to abnormal information processing by the brain^1,2,14^. Studies have associated the development and establishment of schizophrenia with dysfunctions in neurotransmitter systems and oligodendrocytes^15,16^. Oligodendrocytes are cells that wrap around axons in specialized layers of cell membrane to form the myelin sheath. This sheath is important for electrical insulation, increasing axonal conduction velocity, and consequently, neural processing speed^17^.

The oligodendrocyte lineage is composed of a series of developing cells that progressively mature into myelinating postmitotic cells from progenitor cells (OPCs), in a process marked by morphological changes and sequential expression of stage-specific markers, such as 2′,3′-cyclic-nucleotide-3-phosphodiesterase (CNPase), myelin basic protein (MBP) and proteolipid protein (PLP)^18^. This process is characterized by distinct and serial phenotypic stages, such as OPCs, preoligodendrocytes, immature (or pre-myelinating) OLs and mature (or myelinating) OLs ^18^. Extrinsic molecules, such as growth factors, cytokines, hormones and neurotransmitters are fundamental in the regulation of OL maturation^19^.

Neural detachment, as well as other changes of the white matter in schizophrenia, appear to be related to abnormal oligodendrocytes. These alterations include changes in number, spatial distribution and variations in the morphology of these cells^20^. Furthermore, gene expression and proteome analyses suggest a dysregulation of oligodendrocyte and myelin related genes and proteins in patients with schizophrenia^15^. Neuropathological and neuroimaging studies have also found abnormalities and degeneration related to OLs, especially in the frontal and temporal lobes and in the corpus callosum of schizophrenia patients^21^. Moreover, studies have suggested that oligodendrocytes may be a target of antipsychotics^16,21,22^. Thus, studying the effects of these drugs on oligodendrocytes may contribute to the improvement of current treatments and to the development of new therapeutic approaches.

The elucidation of the pathophysiology and the development of more effective treatments are the main challenges in schizophrenia^1^. Therefore, proteomic techniques such as shotgun mass spectrometry have been used in research aiming to identify biomarkers and pathways related to schizophrenia^23^. One substantial goal in functional proteomics is to globally profile changes in protein expression of biological systems in response to different conditions, such as a disease or after drug treatment. Identifying and quantifying proteins in such highly complex mixtures requires accurate and sensitive assays^24^. One possibility to characterize and compare complex mixtures in a concentration-dependent manner is the use of chromatographic separation in combination with electrospray ionization mass spectrometry (ESI–MS)^25^. This approach, combined with label-free quantitation methods, is an extremely useful tool in large-scale proteomic analyses of complex biological samples, such as cell lines, bodily fluids and tissues^26,27^.

In this study, we analyzed the proteome of a matured human oligodendrocyte cell line (MO3.13 cells) treated with haloperidol (a first-generation antipsychotic), clozapine (a second-generation antipsychotic) and clozapine + d-serine, by a nanoESI-LC–MS/MS label-free analysis. MO3.13 cells are an immortalized hybrid cell line that expresses phenotypic characteristics of primary oligodendrocytes^28^. Techniques such as differentiation and maturation of cell lines and progenitor cells are crucial for the improvement and development of in vitro models that better represent the in vivo cells, and are also practical to the scientific community^29^. Therefore, we used a maturation model of MO3.13 cells, previously described in Seabra et al.,^29^**,** in order to study the effects of antipsychotics on the proteome of oligodendrocytes. The matured cells are similar to pre-myelinating OLs, with increased PLP expression in comparison to the initial MO3.13 cells. From this, we identified proteins and biochemical pathways that were affected by the presence of these drugs, investigating their influence on oligodendrocyte function, which may prove to be important for the development of more effective treatments for schizophrenia.

## Results and discussion

**Figure 1:**
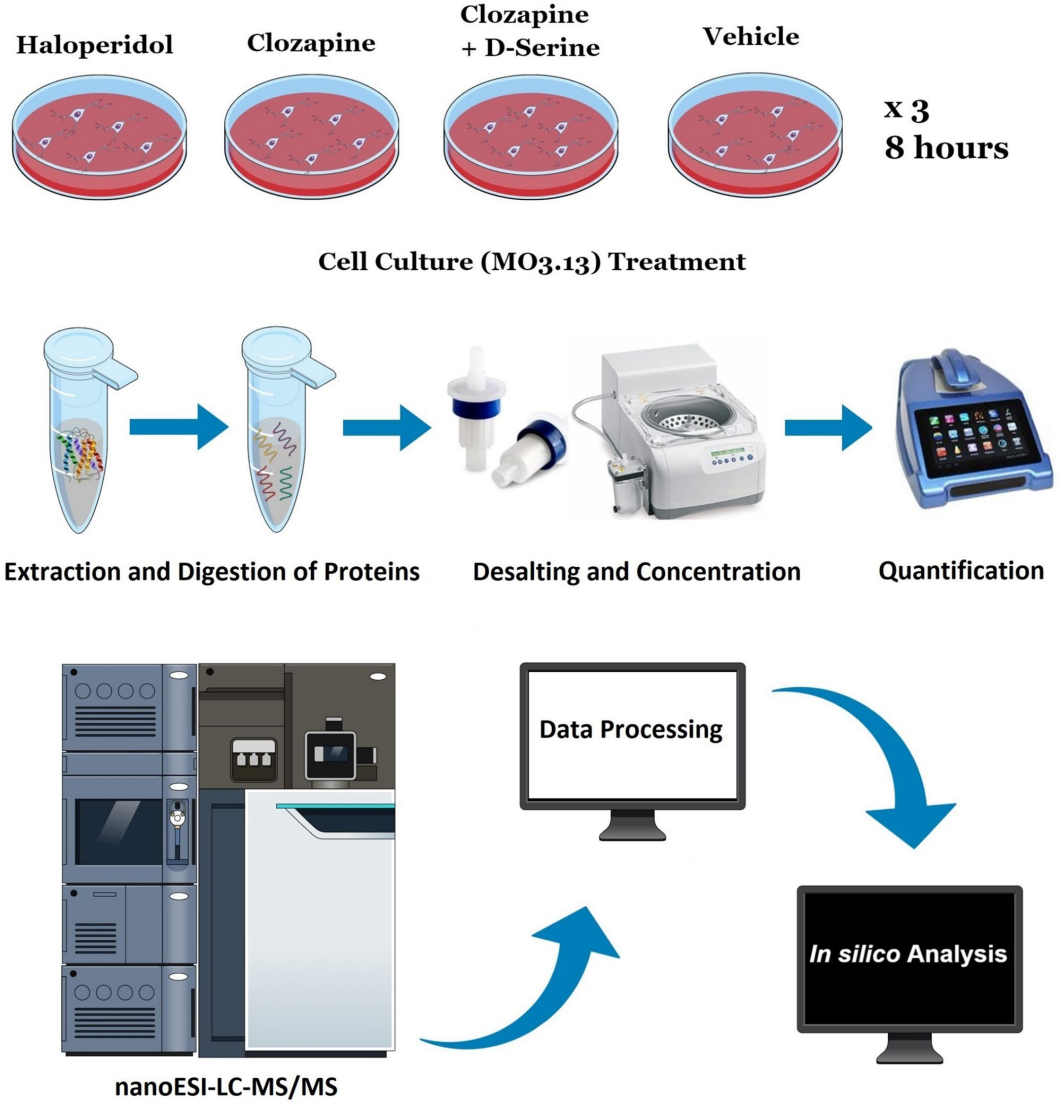
Experimental steps from cell culture to in silico analysis. This figure was created using the Mind the Graph platform (https://www.mindthegraph.com).

Using a three-fraction HDMS^E^, we identified a total of 1677 proteins. After applying stringent searching criteria (as described in 4.4. section) followed by a label-free method, we considered 1,427 proteins as the proteome of matured MO3.13 cells, with which we performed all systems biology analyses that follows (Fig. 1).

**Figure 2:**
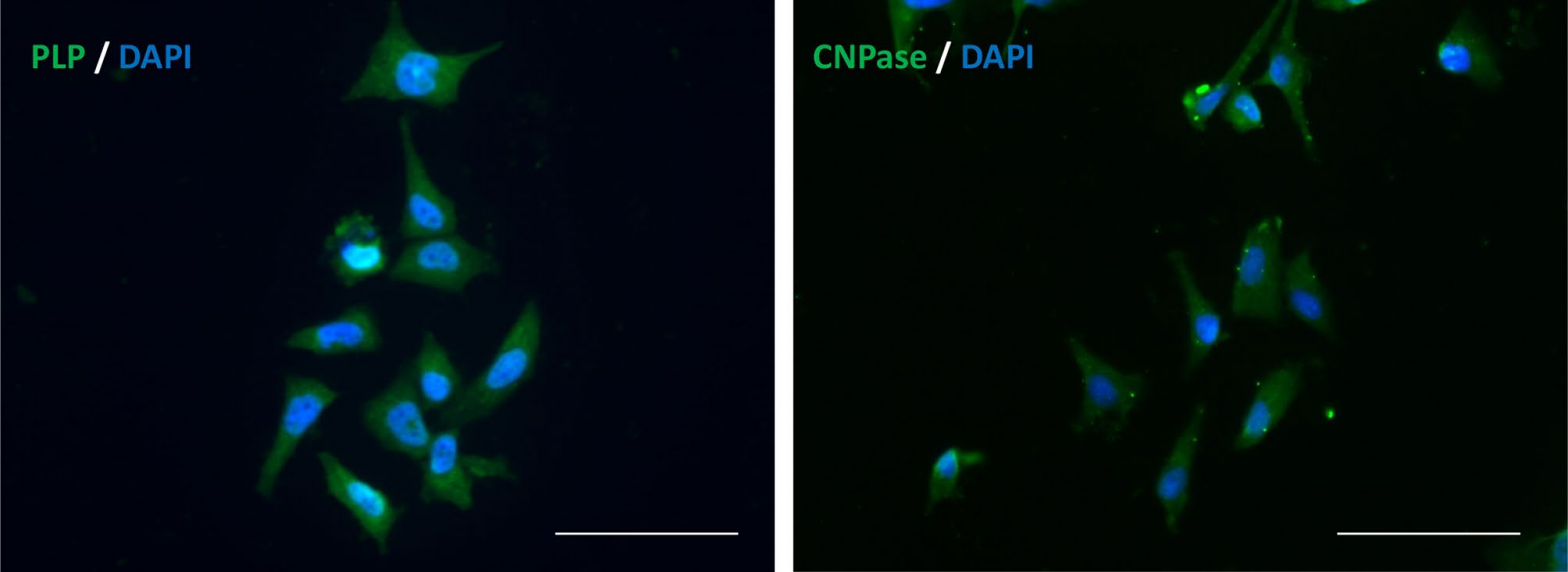
MO3.13 cells labeled with antibodies for PLP (left) and CNPase (right) (in green) and DAPI (in blue), after 15 days in maturation media. Scale bar: 200 µm.

**Figure 3:**
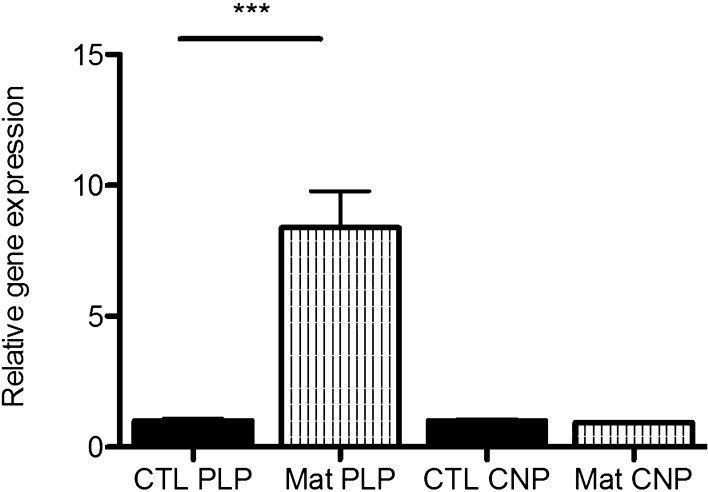
Relative gene expression of PLP and CNPase in MO3.13 cells after maturation protocol (Mat), compared to cells cultured in non-maturing media (CTL). These data were obtained by RT-qPCR and normalized to the expression of ACTB gene. Relative quantification value of each target gene was analyzed using a comparative CT method^123^. Statistical significance was determined by Student t-test, ***p < 0.001 compared to CTL group.

Figure 2 shows the MO3.13 cells labeled with anti-PLP (left) and anti-CNPase (right) antibodies after 15 days in maturation media. We also verified an eightfold increase in PLP expression in the cells by RT-qPCR (Fig. 3) after maturation protocol, while CNPase levels showed no significant changes. PLP is a marker for mature OLs, while CNPase is expressed both by immature and mature OLs^18^. CNP is highly expressed in maturing OLs to avoid myelin compaction in early stages^30^. In this context, there is a balance on CNP and MBP levels, as the latter is more expressed in myelinating OLs^31^. Thus, in advanced stages of maturation, CNP returns to basal levels allowing myelin ensheathment, in which MBP participates. Myelin sheath formation and compactation do not occur in our cell model, and this might be related to unchanged CNPase levels. Considering the conditions our cells were cultured in, we believe our cells have matured, since they might be similar to pre-myelinating OLs as proposed by Dawson et al.^32^.

The number of differentially expressed proteins and their regulation are displayed in Table 1. Data were analyzed using in silico systems biology tools in order to identify biochemical pathways and biological processes in which differentially expressed proteins are involved in, as well as their interaction networks.

**Table 1:** Number of differentially expressed proteins obtained from Hi3-based shotgun proteomic analyses per treatment (compared to vehicle).

Treatment	Modulated proteins^a^	Upregulated	Downregulated
Clozapine	173	85	88
Clozapine + d-serine	84	45	39
Haloperidol	467	318	149

^a^Include upregulated and downregulated proteins with ANOVA p-value < 0.1

**Figure 4:**
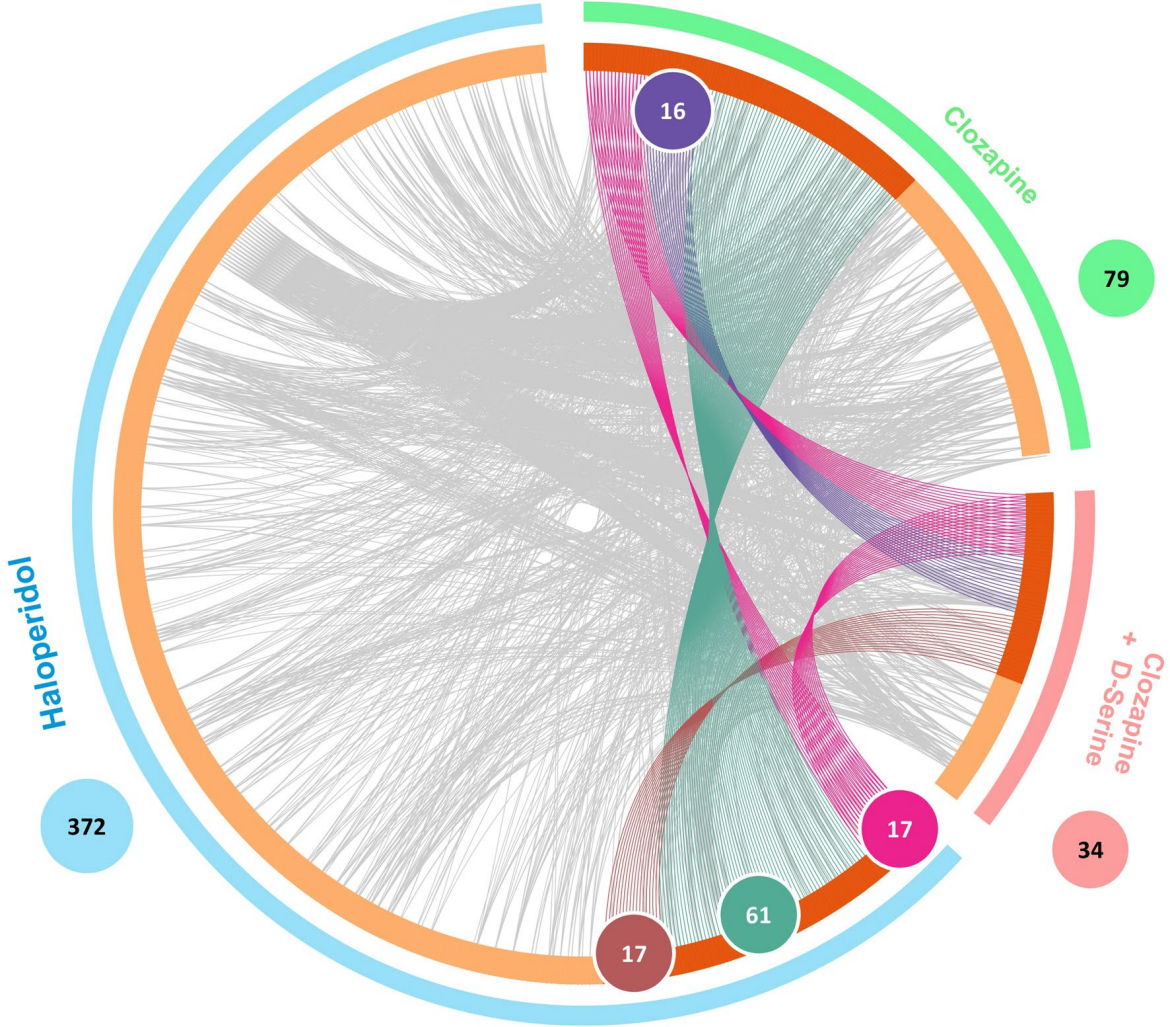
Chord diagram of the number of modulated proteins (ANOVA p-value ≤ 0.1) in matured MO3.13 cells treated with haloperidol, clozapine or clozapine + D-serine. This figure was made using Metascape^122^.

**Figure 5:**
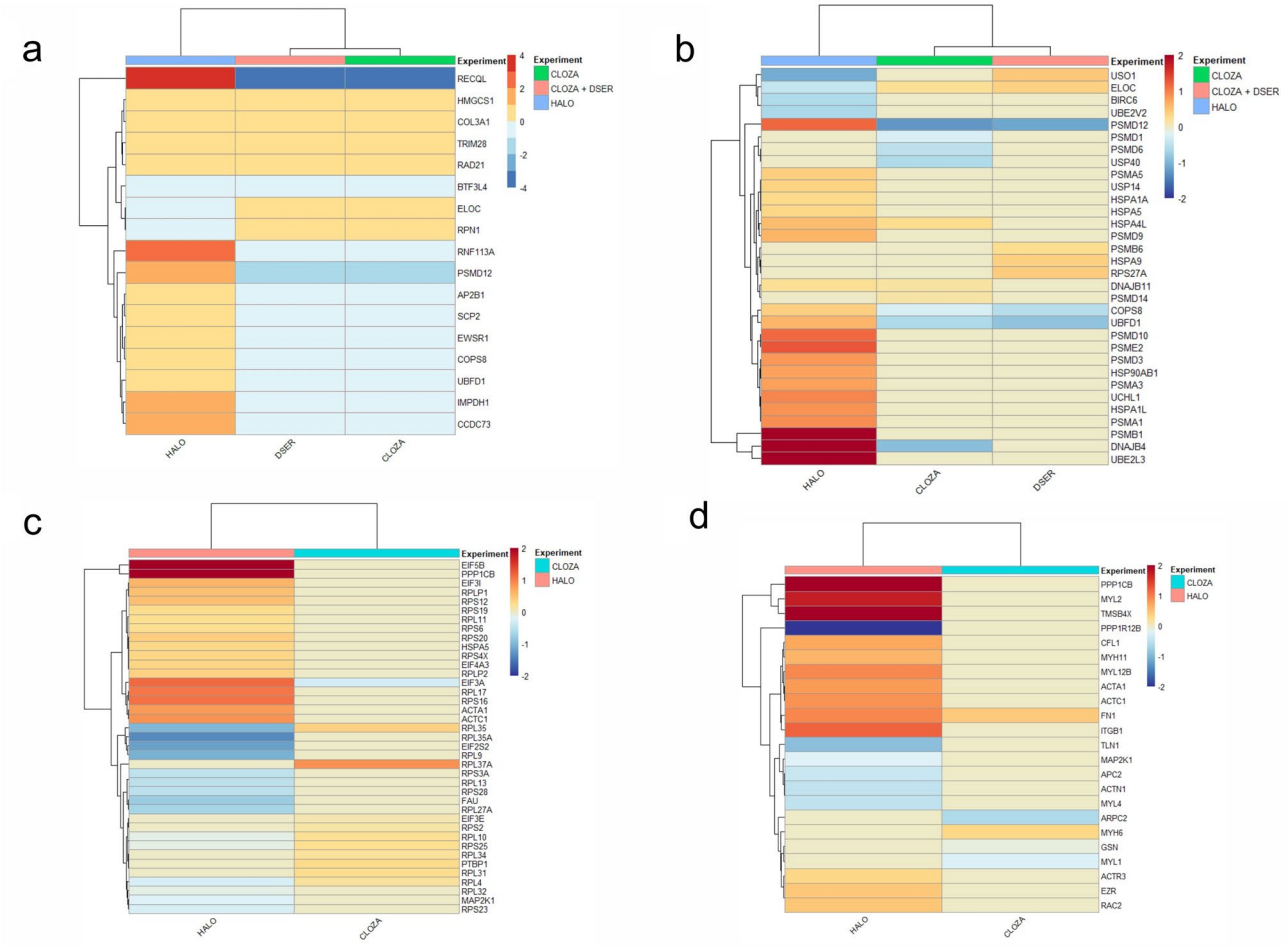
Differentially expressed proteins in common for MO3.13 cells treated with haloperidol, clozapine and clozapine + d-serine (**a**). The UPS-related proteins modulated by those treatments are show in (**b**). Haloperidol and Clozapine also modulated proteins related to eIF2 signaling (**c**) and to the actin cytoskeleton (**d**). Proteins in shades of red are upregulated, while the ones in shades of blue are downregulated. This figure was made using Rstudio (version 1.2.5033)^123^, R (version 3.6.2)^124^, Pheatmap package (version 1.0.12)^125^.

We found an overlap (Fig. 4) and treatment-specific modulation of the proteome by treatments. The 17 proteins modulated by all three treatments are displayed in Fig. 5a. Besides altering proteins involved in ubiquitination, proteasome degradation and lipid metabolism, all three treatments modulated DNA damage repair-related proteins, such as RECQL (ATP-dependent DNA helicase Q1), TRIM28 (Tripartite motif containing 28), RAD21 (Double-strand-break repair protein rad21 homolog) and RNF113A (Ring finger protein 113A). Therefore, these processes seem to be relevant in the oligodendrocyte response to antipsychotics.

### Common Targets—Proteins related to ubiquitin–proteasome system (UPS)

Among the proteins modulated by all treatments are PSMD12 (26S proteasome non-ATPase regulatory subunit 12), UBFD1 (ubiquitin family domain containing 1) and COPS8 (COP9 signalosome complex subunit 8), all related to ubiquitination and degradation by proteasome. Other proteins related to the ubiquitin–proteasome system (UPS) were also modulated by these antipsychotics (Fig. 5b). The UPS plays a role in diverse cellular processes, such as protein labeling for degradation, vesicle and protein transport, and membrane receptor recycling^33^. Thus, if this mechanism has a functional or structural impairment, proteolytic activity is impaired, which may lead to an accumulation of damaged/dysfunctional proteins. This has already been associated with the pathogenesis and phenotypic characteristics of neurodegenerative diseases, including Alzheimer's^34^ and Parkinson's^35^.

Proteasomes also contribute to aspects of neural function that are abnormal in schizophrenia, including neurodevelopmental maintenance, dendritic morphology, energy homeostasis, neurotransmitter synthesis, receptor recycling and cytokine production and activation^36–40^. Several changes in the UPS have been related to schizophrenia through studies involving *postmortem* brains of patients. These studies have found changes in the levels of ubiquitinated proteins^41,42^, the expression of ubiquitinating, deubiquitinating and conjugating enzymes^42–47^ and a reduction in free ubiquitin levels^47^.

As discussed, changes in protein transport and turnover, and ubiquitination-related signaling pathways can lead to brain tissue abnormalities that may contribute to neurotoxicity and functional impairment^41,48^. Therefore, elements of the UPS may act as potential pharmacological targets^41^. Our data are in agreement with this hypothesis and suggest that the modulation of proteins involved in the UPS is a common effect of typical and atypical antipsychotics in oligodendrocytes.

### Common targets—proteins related to lipid metabolism

Proteins related to lipid metabolism were also modulated by the three treatments, among them: SCP-2 (sterol carrier protein 2) (ANOVA p-value: 0.0695 for haloperidol; 0.0663 for clozapine; 0.0507 for clozapine + d-serine) and HMG-CoA synthase (hydroxymethylglutaryl-CoA synthase) (ANOVA p-value: 0.0629 for haloperidol; 0.0017 for clozapine; 0.0708 for clozapine + d-serine). SCP-2 binds and transports lipid ligands such as long chain fatty acids and their CoA thioesters^49^, endocannabinoids^50^ and phospholipids^51^. Thus, SCP-2 is an important component in maintaining lipid homeostasis. Meanwhile, HMG-CoA synthase catalyzes the condensation of acetyl-CoA with acetoacetyl-CoA to form 3-hydroxy-3-methylglutaryl-CoA (HMG-CoA), an intermediate in cholesterol synthesis and ketogenesis^52^

Dysfunctions in lipid homeostasis can affect neural function, synaptic transmission, cell signaling, myelination and oligodendrocyte biology^53^, contributing to white matter dysfunction and disturbances in neural connectivity^54^. Therefore, changes in lipids have been associated with the pathophysiology of schizophrenia. Specifically, alterations related to fatty acids and ketone bodies^55,56^, phospholipids^57,58^ sphingolipids^59^, total lipids, triglycerides and cholesterol esters were observed in schizophrenia patients^60–62^.

Regarding antipsychotics, studies have suggested that these drugs may compensate for or reverse lipid pathway dysregulation related to the early stage of the disorder^55,59,63^. Furthermore, fatty acid metabolism and cholesterol biosynthesis have been identified by gene expression studies as key elements in antipsychotic treatment response^64,65^. One hypothesis proposes that the modulation of cholesterol and the proportion of polyunsaturated and saturated fatty acids by antipsychotics may affect neural membrane fluidity, resulting in changes in neural connectivity^65^, which may be related to therapeutic effects.

In our data, HMG-CoA synthase, responsible for HMG-CoA synthesis, is upregulated. The up-regulation of the hydroxymethylglutaryl-CoA synthase 1 gene (HMGCS1) has been previously described by studies evaluating the effect of clozapine, olanzapine, haloperidol, chlorpromazine and risperidone on human glioma strains^64^ and epithelial cell retinal pigment (ARPE-19)^65^. The up-regulation of this enzyme may contribute to the synthesis of cholesterol, an important component of the myelin sheath and a key element of oligodendrocyte metabolism.

### Haloperidol and clozapine—canonical pathways

Signaling by Rho GTPase proteins is evident in haloperidol treatment, while processes involved in actin cytoskeleton and eIF2 (eukaryotic initiation factor 2) signaling are modulated by both drugs. The proteins that were modulated by these treatments and are involved in eIF2 signaling and actin cytoskeleton are shown in Fig. 5c,d, respectively.

eIF2 is a transcription initiation factor critical for protein synthesis^66^. Furthermore, eIF2B plays a key role in maintaining the viability of oligodendrocytes, and its impaired activity can lead to a dysfunctional stress response^66^ in thes cells. eIF2B-related mutations are associated with the evanescent white matter disease, which causes severe loss of oligodendrocytes and astrocytes in early life^67^. We found that haloperidol treatment contributed to the downregulation of this pathway, while clozapine treatment upregulated the pathway. Thus, our findings suggest that typical and atypical antipsychotics may have distinct effects on protein synthesis, as well as on the maintenance of oligodendrocyte viability mediated by eIF2 signaling, in agreement with another study of our group^68^.

Actin cytoskeleton signaling was also modulated by haloperidol and clozapine treatments, being upregulated in both cases. Actin is critical in stabilizing and maintaining cell morphology and its dynamics underlies several central cellular processes, such as cell motility and intracellular protein traffic^69^. In the nervous system, this process is fundamental in maintaining the morphology and density of dendritic spines^70^. Furthermore, actin cytoskeleton regulation has been associated with schizophrenia^71–74^. Alterations in the expression of proteins involved in the regulation of actin polymerization and dynamics were observed in the dorsolateral prefrontal cortex (DLPFC)^72^ and in the anterior cingulate cortex (ACC) of schizophrenia patients^71^. Thus, it is possible that haloperidol and clozapine treatments help to modulate actin cytoskeleton signaling in oligodendrocytes, which might be related to their therapeutic effects.

### Haloperidol

**Figure 6:**
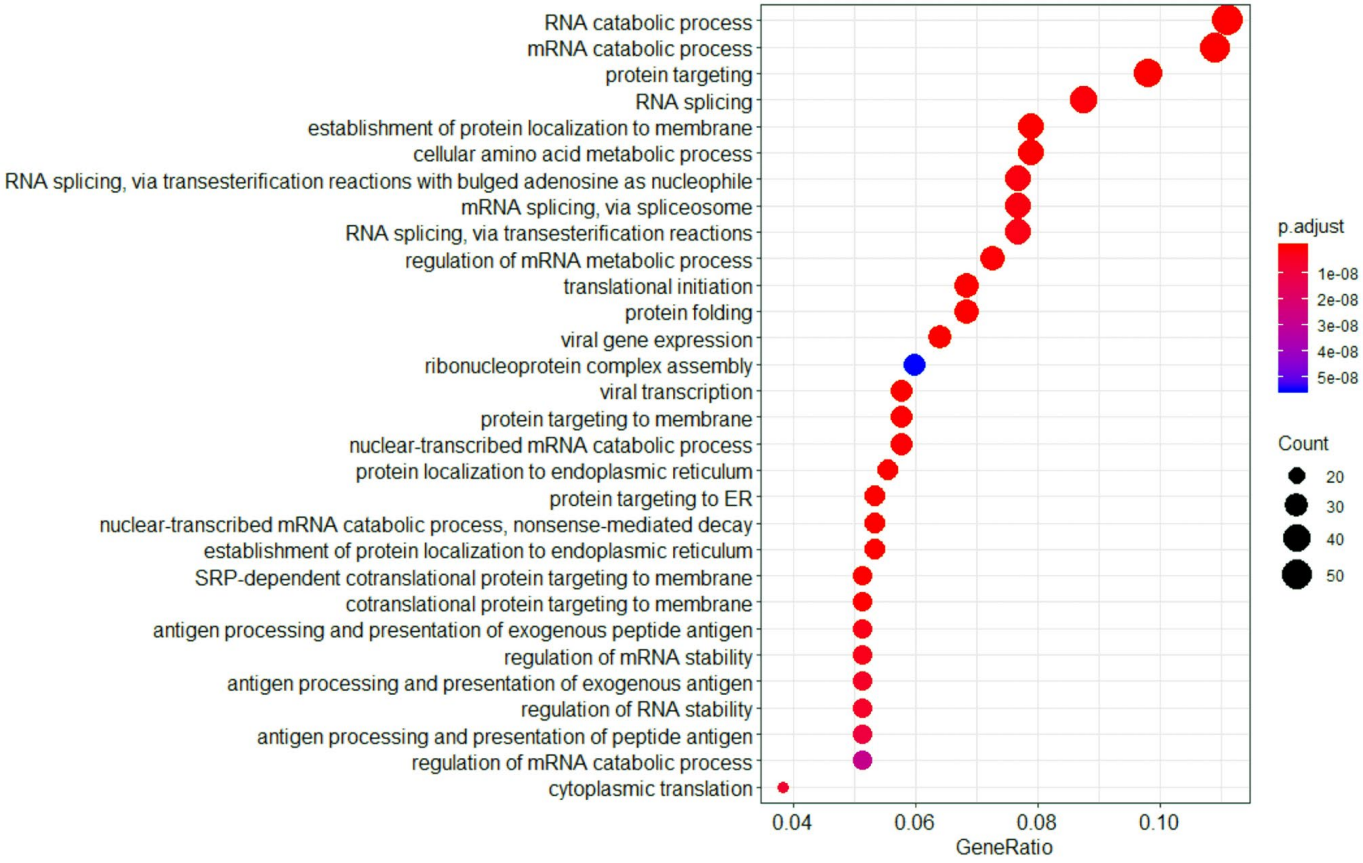
Dotplot of biological processes modulated by haloperidol treatment (TOP30), with protein count and adjusted p-value. This figure was made using R ClusterProfiler (version 3.14).

Haloperidol treatment affected, among others, pathways involved in amino acid metabolism, protein targeting and signal transduction (by Rho GTPases, for example). This treatment also modulated several proteins related to RNA splicing (via both transesterification reactions and spliceosome), and RNA metabolism (especially catabolism) and stability, as show in Fig. 6. A previous study of our group has shown that chlorpromazine, haloperidol, quetiapine and risperidone treatments affected proteins of spliceosome machinery in MO3.13 cells, especially belonging to nuclear heterogeneous ribonucleoprotein (hnRNP) family^68^.

Several studies have identified global changes in alternative splicing in schizophrenia by comparing the transcriptomes of tissues from patients to healthy controls^75^. BICD2 and DLG3 exons, for example, were differentially expressed in brain and blood samples of schizophrenia patients^76^. Other genes that present abnormal splicing patterns in *postmortem* brain include DISC1^77^, ERBB4^78^, NRG1^79^ e NRG3^80^. Detailed data about alterations in alternative splicing in schizophrenia and other psychiatric disorders can be found in Morikawa and Manabe^81^ and Reble et al.^75^ reviews. Therefore, RNA splicing can be an important target for further studies in schizophrenia, considering the alterations previously described in literature and the modulation of related proteins by haloperidol and other antipsychotic treatments.

Furthermore, haloperidol treatment modulated proteins involved in the synthesis of selenocysteine, an important amino acid found in some proteins, conferring them specific properites; these proteins are often involved in cellular protection against oxidative stress, such as glutathione peroxidase^82–84^. On the other hand, there is evidence to suggest an increase in oxidative stress markers associated with antipsychotic use, especially typical drugs^85–88^. Deficits in defense mechanisms against free radicals were also related to the development of tardive dyskinesia, a notable extrapyramidal side effect of typical antipsychotics^88^. Tying these data together, we hypothesize that the impairment of selenocysteine synthesis may affect the functioning of enzymes involved in cellular protection against oxidative stress, contributing to an oxidative stress scenario, which might in turn related to the development of tardive dyskinesia.

In addition to the aforementioned proteins, several others modulated by haloperidol are involved in Rho-GTPase signaling, important to regulate the actin cytoskeleton. *Postmortem* studies reported a decreased expression of Rho-GTPase proteins in schizophrenia patients^74,89^. Rho GTPase Cdc42 mRNA levels, involved in actin polymerization regulation^90,91^, are decreased in the DLPFC^74^. Furthermore, a dysregulation of the RhoA pathway (crucial in controlling brain size and connectivity) is considered to be a risk factor for autism and for the development of schizophrenia^92^. In our study haloperidol upregulated RhoA and Cdc42 and downregulated RhoGDI, strengthening the previously found data. Therefore, this drug may have a role in regulating Rho-GTPase signaling in oligodendrocytes.

### Clozapine

**Figure 7:**
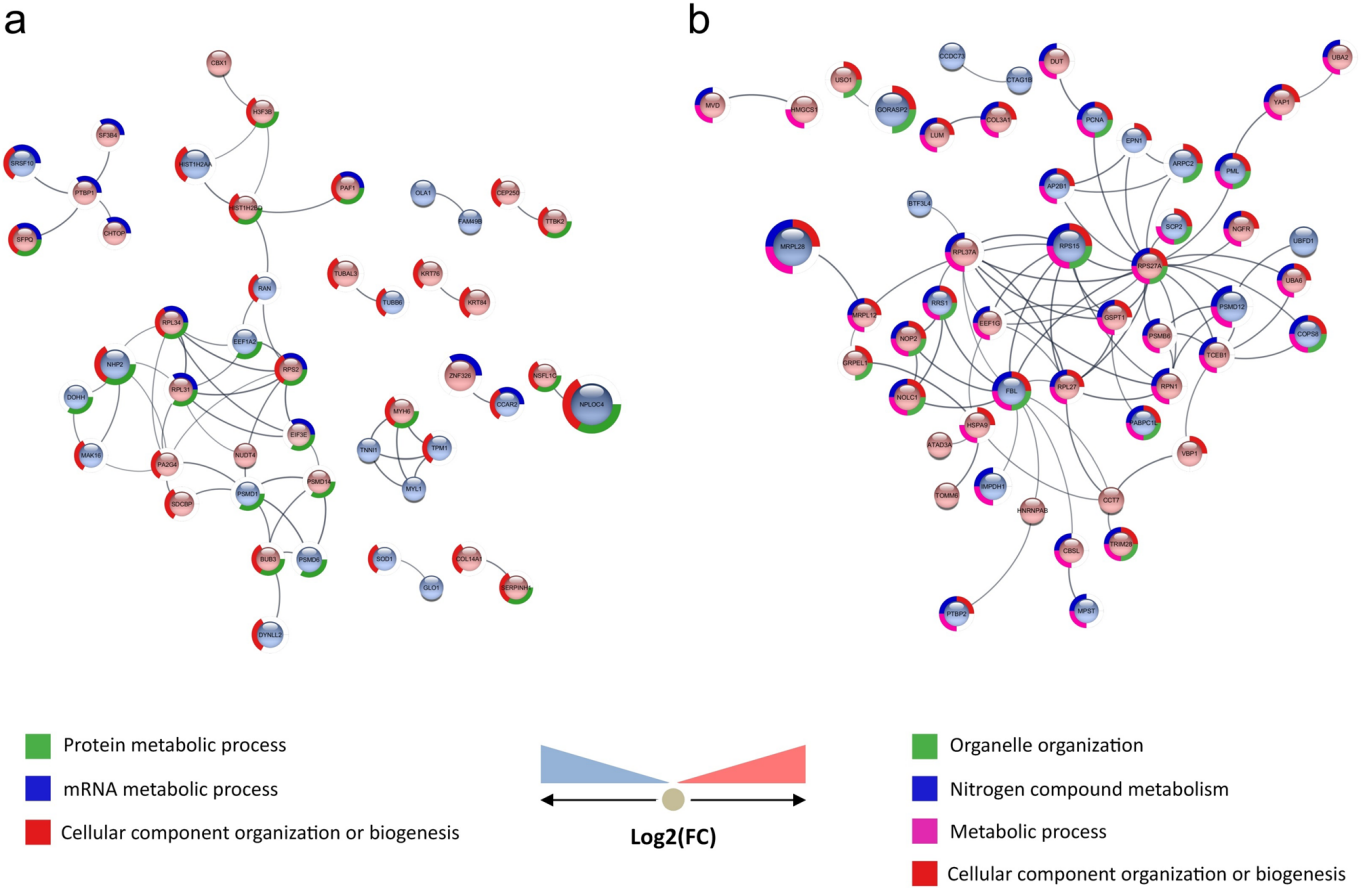
Interactome of proteins that were differentially expressed exclusively by clozapine treatment (**a**) and by clozapine + d-serine treatment (**b**). Known interactions between proteins are represented by lines connecting the nodes. The darker the line, the greater the degree of confidence about the interaction, calculated from evidence found in literature and databases. This figure was made using Cytoscape (version 3.7.2)^126^ and String app (version 1.5)^127^, with a minimum required interaction score of 0.700. (**a**) Blue bar indicates involvement in mRNA metabolism, red bar indicates involvement with in the organization or biogenesis of cellular components, and green bar indicates involvement in cellular protein metabolism. (**b**) Red bar indicates association with the organization or biogenesis of cellular components (especially organelles, such as ribosomes). Pink bar indicates participation in metabolic processes, both catabolic and anabolic. Blue bar indicates participation in the metabolism of nitrogenous compounds. Green bar indicates a relation to organelle organization.

Clozapine treatment modulated proteins related to metabolism, gene expression regulation, RNA processing, axonal guidance, signal transduction and cellular response to stress, among others. The interactome of proteins modulated exclusively by clozapine treatment highlighted some processes, such as the organization or biogenesis of cellular components and mRNA metabolism (Fig. 7a). Proteins involved in protection against oxidative stress, such as glutaredoxin, superoxide dismutase (ANOVA p-value: 0.07893) and thioredoxin (TRX) (ANOVA p-value: 0.0891) were also affected.

TRX is a ubiquitous protein with oxide reductase activity^93^, associated with cognitive deficits in schizophrenia^94,95^. Increased TRX levels in serum have been observed in first-episode, psychotic-naïve patients, compared both to antipsychotic-treated and control subjects^96^. Thus, TRX serum levels are higher during acute psychotic episodes, tending to decrease after the remission of symptoms^97^. According to our data, TRX was downregulated by clozapine in oligodendrocytes, corroborating the previous findings that decreased levels of this protein are associated with antipsychotic treatment. However, further investigations are needed to understand the outcome for oligodendrocyte biology.

Furthermore, some proteins related to mitochondrial function and dynamics were affected by clozapine, such as cytochrome c oxidase, subunit 5B (upregulated; ANOVA p-value: 0.0752), acyl-CoA dehydrogenase of mid-chain fatty acids (downregulated; ANOVA p-value: 0.0031), TOM34 (outer mitochondrial membrane translocase 34; downregulated; ANOVA p-value: 0.0104) and TIM13 (inner mitochondrial membrane translocase 13; downregulated; ANOVA p-value: 0.0951). This treatment also modulated proteins related to the organization or biogenesis of cellular components, such as organelles, in MO3.13 cells.

Mitochondrial dysfunctions in schizophrenia include metabolic, enzymatic, anatomical and genetic abnormalities^98^. Studies have detected a reduced number of mitochondria in oligodendrocytes of the ACC^99,100^ and a reduction in mitochondrial size in caudate oligodendrocytes^101^. These alterations may affect the energy supply of these cells, and consequently the myelination process^98^. Antipsychotics, in turn, affect mitochondrial number, size and function, depending on the brain region, dose, length of use and routine of administration^98^. Thus, we hypothesize that the modulation of proteins involved in mitochondrial function and dynamics, as well as organelle organization and biogenesis, may modulate mitochondrial biology, both structurally and regarding the number of organelles. This can affect processes such as myelination and energy supply of neuronal axons, important functions of oligodendrocytes for maintaining neural connectivity.

### Clozapine + d-Serine

Clozapine + d-serine treatment modulated proteins related to metabolism, signal transduction, immune system and transport, among others. Some processes were shown in the interactome of affected proteins, such as the organization or biogenesis of cellular components (especially organelles), metabolic processes and the metabolism of nitrogenous compounds (Fig. 7b), a predominant target of modulation to this treatment.

Some studies have suggested a relationship between changes in nitrogen metabolism and symptomatic worsening and remission in schizophrenia^102,103^*.* A reduction in plasma nitrogenous amino acid levels has been observed in patients, especially during acute attacks, and an increase is seen with symptom remission^103^. Moreover, increased levels of N-acetylglutamine and reduced levels of N6-acetyl-l-lysine were observed in schizophrenia patients’ serum in comparison to control subjects^104^. Reduced concentrations of N-acetyl aspartate in the prefrontal cortex of chronic patients^105^ were also reported.

Other studies have found an increase in plasmatic nitric oxide (NO) levels in schizophrenia patients compared to control subjects^106, 107^. Furthermore, patients in the acute psychotic phase present extremely high levels of 3-nitrotyrosine (a marker of peroxynitrite production) on plasmatic proteins^108^. Therefore, peroxynitrite and other reactive nitrogen species, including NO and its metabolites, may be related to schizophrenia^106–108^. Thus, considering the expressive modulation of proteins involved in the metabolism of nitrogenous compounds, it is possible that co-treatment with clozapine + d-serine contributes to the normalization of the metabolism of these compounds. This hypothesis can be tested in future studies focusing on nitrogenous compounds levels and metabolism.

Decreased levels of d-serine have been reported in blood and cerebrospinal fluid (CSF) of schizophrenia patients^12, 109–111^, as well as disturbance of its metabolizing enzymes^112^. This amino acid administered in combination with usual antipsychotics was found to be more effective in relieving symptoms of schizophrenia, when compared to the administration of antipsychotics alone^11, 13^. d-Serine also seems to be related to neurophysiologic changes induced by cognitive training in schizophrenia, with increased levels being positively correlated with improvements in verbal learning and global cognition^113^. Further, good responders to clozapine presented an increase in d-serine plasma levels^114^. Therefore, the elucidation of proteins and pathways affected by d-Serine co-administration with antipsychotics is important to understand the mechanisms that might be related to symptom improvements associated to d-Serine use.

## Conclusion

From this study, we identified proteins and biochemical pathways affected by antipsychotic treatment on oligodendrocytes through *shotgun* proteomics, using nanoESI-LC label-free shotgun mass spectrometry. Some affected proteins and processes were common to haloperidol, clozapine and clozapine + d-serine treatments. These were mainly related to ubiquitination, proteasome degradation, lipid metabolism and DNA damage repair. Clozapine and haloperidol also modulated proteins involved with the actin cytoskeleton and eIF2 signaling. Haloperidol treatment affected, among others, proteins involved in Rho GTPase signaling and in the synthesis of selenocysteine. Clozapine treatment modulated proteins related to metabolism, protection against oxidative stress (such as TRX) and organization or biogenesis of cellular components, among others. In turn, metabolic processes, especially the metabolism of nitrogenous compounds, were a predominant target of modulation of clozapine + d-serine treatment. With this data, we sought to contribute to the understanding of the biochemical and molecular mechanisms involved in the action of these drugs on oligodendrocytes and their possible implications in schizophrenia. Therefore, we aim to contribute to future studies focusing on the pathways and proteins highlighted by this work, and to studies dedicated to improving current treatments and developing new therapeutic approaches.

## Methods

### Cell culture and treatments

MO3.13 cells were initially cultured in Dulbecco’s modified eagle medium (DMEM) (Sigma-Aldrich), containing 0.5% penicillin/streptomycin (Gibco) and 10% fetal bovine serum (FBS) (Gibco), renewed every 2 days, as previously described in Brandão-Teles et al. (2017)^115^. For maturation, cells were cultured in 6-well plates (Corning, Inc.) containing DMEM (Sigma-Aldrich) supplemented with 0.5% N2 (Gibco), 0.5% B27 (Gibco), PDGFα (alpha-type platelet-derived growth factor) (10 ng/ml) (Thermo Fisher Scientific), 0.1% penicillin/streptomycin (Gibco) and triiodothyronine (T3—30 ng /ml) (Sigma-Aldrich) at 37 °C in 5% CO_2_. The medium was renewed every 3 days for a maturation period of 15 days. The procedures for thawing, culturing, counting, passaging, maturation and collection of cells are described in Seabra et al.^29^.

The cells were treated, in triplicate, with: (a) 50 μM clozapine (Cristália); (b) 50 μM haloperidol (Cristália); (c) vehicle solution (0.01 M HCl); or (d) 50 μM clozapine + D-serine (Sigma). After 8 h, the cells were collected in PBS (1 ×; Sigma-Aldrich) and scraped with a plastic scraper, according to Seabra et al.^29^. Afterwards, the samples were centrifuged at 200 × *g* for 5 min (FANEM 206BL Centrifuge), the supernatant was removed and the cellular pellets were stored at − 80 °C for later analyses.

### Sample preparation

Cellular precipitates derived from the four conditions (clozapine, haloperidol, clozapine + d-serine or vehicle) in biological triplicate were homogenized in 100 μl of lysis buffer (containing 6 M urea [Sigma-Aldrich], 2 M thiourea [Synth], 10 mM dithiothreitol [DTT], 0.1 mM sodium pervanadate and 10 μL of 10 × protease inhibitor [Roche]). Then, they were vortexed and incubated for two hours at 37 °C. After incubation, samples were diluted ten times with 20 mM TEAB (triethylammonium bicarbonate buffer), pH 7.5 (Sigma-Aldrich) and sonicated on ice (Ultrasonic Homogenizer 4,710 Series, Cole-Parmer Instrument Co.) for 20 s (pulser on, duty cycle: 25%, micro-tip limit: 1.5). Next, samples were alkylated by incubation with 100 μl of 20 mM iodoacetamide (IAA) (Amersham Biosciences) for 20 min in the dark at room temperature (RT). Trypsin (sequencing grade; Sigma-Aldrich/Promega) was used for digestion, (1:50 enzyme: substrate), with overnight incubation (12–16 h) at 37 °C. After incubation, the reaction was stopped with formic acid (Sigma-Aldrich) (final concentration 5% v/v) and incubated for 5 min at RT. Subsequently, the samples were centrifuged for 45 min at 4 °C and 20,850×*g* (5430R Centrifuge—Eppendorf) and the supernatants were transferred to low-binding microtubes (Axygen).

Desalting and concentration of the peptides were done with HLB cartridges (Oasis-Waters) according to Brandão-Teles et al.^115^. Briefly, HLB (Oasis-Waters) cartridges were activated with 1 mL of 100% methanol (Merck), followed by 1 mL of 100% acetonitrile (ACN) (Merck), and equilibrated with 2 mL of 0.1% trifluoroacetic acid (TFA) (PerkinElmer). Then, samples were slowly added to the cartridges over two additions to load the full volume. The cartridge was washed twice with 2 mL of 0.1% TFA (PerkinElmer) and the peptides were eluted with 2 mL of 70% ACN/0.1% TFA. Then, the samples were concentrated (Eppendorf Concentrator Plus) and reconstituted in 20 mM ammonium formate, pH10. Next, the concentration of peptides was determined (DeNovix—DS11 Spectrophotometer) and samples were diluted to a final concentration of 1 μg/μL with 20 mM ammonium formate, pH10.

### NanoESI-LC–MS/MS

The proteomic analyses were performed using a two-dimensional Acquity M-Class nanoUPLC system (Waters Corporation, Milford, MA), coupled to a Synapt G2-Si spectrometer (Waters Corporation, Milford, MA). The MS and MS/MS data were obtained using data-independent acquisition (DIA) and ion mobility separation. The peptide samples derived from the four conditions in biological triplicate (in 20 mM ammonium formate, pH10) were analyzed using an HDMS^E^ (high-definition data-independent mass spectrometry) label-free method, with three fractions of 54 min in reverse-phase chromatography (steps of 13.7%, 18.4% and 50% of acetonitrile in the first dimension; and a linear gradient of 7–40% ACN in the second dimension). In the first-dimension, peptides were loaded onto an M-Class BEH C18 Column (130 Å, 5 μm, 300 μm × 50 mm, Waters Corporation, Milford, MA, United States), and a nanoACQUITY UPLC HSS T3 Column (100 Å, 1.8 μm, 75 μm × 150 mm, Waters Corporation, Milford, MA, United States) was used for the second dimension. Injections were performed using a nano-electrospray ionization source in positive ion mode (nanoESI (+), with a NanoLock-Spray (Waters, Manchester, United Kingdom) ionization source. A solution of [Glu1]-Fibrinopeptide B (Glu-Fib; Human) was used as the lock mass, and was sampled every 30 s. Biological triplicates were performed following the processing software specifications (Progenesis QI for Proteomics 3.0, Waters).

### Data processing

MS and MS/MS spectra were processed with database search and a processing software. Progenesis QI for Proteomics 3.0 (Waters) was used to perform qualitative and quantitative, label-free analyses. In this process, the following parameters were considered: 5 as maximum peptide charge, maximum protein mass of 600 kDa, a maximum of 1 missed cleavage, at least 2 fragments per peptide, at least 5 fragments per protein, and at least one peptide per protein, with an FDR of < 1% and a mass error cutoff of 20 PPM. Hi-3 was used as the quantitation method, in which protein quantitation is based on the average integrated signal intensity of the top 3 most intense peptides^116, 117^. Cysteine carbamidomethylation and methionine oxidation were considered as fixed and variable modifications, respectively. The database used was reviewed human UNIPROT databank (version 2018/10). In the final list of proteins, identifications with at least one unique peptide were considered for in silico analysis. Quantitation differences that returned a p-value ≤ 0.1 from the ANOVA test were considered differentially expressed between the antipsychotics treated groups (haloperidol, clozapine and clozapine + d-serine) in comparison to the vehicle. The expression analysis was performed considering the three biological replicates available for each experimental condition, and each group as an independent variable.

### In silico analyses

The differentially expressed proteins were classified according to the biological processes in which they were involved using reference databases for human proteins. In order to visualize the network of altered proteins and affected biochemical pathways, we used Ingenuity Pathway Analysis (IPA) 2.4 (QIAGEN Inc.)^118^, which took into consideration the accession of differentially expressed proteins, ANOVA test p value (p ≤ 0.1) and Log2 fold change (considering the ratio between treatment and control). Online software was also used: Reactome (version 69)^119^, STRING (version 11.0)^120^, VENNY (version 2.1)^121^**,** Metascape^122^**,** Rstudio (version 1.2.5033)^123^, R (version 3.6.2)^124^, Pheatmap package (version 1.0.12)^125^, Cytoscape (version 3.7.2)^126^ and String app (version 1.5)^127^. The experimental steps described thus far are shown in Fig. 1.

### Immunocytochemistry

MO3.13 cells were grown in 24-well plates (Corning Inc.) containing glass coverslips. For fixation, each well was incubated with 250 μL 4% paraformaldehyde (PFA) (Sigma-Aldrich)/PBS 1 × solution (Sigma-Aldrich) for 25 min at RT. The cells were washed 3 times with PBS 1 × (Sigma-Aldrich) and the coverslips were incubated with 0.1 M glycine (Sigma-Aldrich) for 20 min at RT (in a wet chamber), followed by a wash with 1 × PBS (Sigma-Aldrich). For the coverslips to be labeled by CNPase (Abcam—Ab28486), the membranes were permeabilized using 0.2% Triton X-100 (Sigma-Aldrich) in PBS 1 × (Sigma-Aldrich) for 2 min, with subsequent washing with 1 × PBS (Sigma-Aldrich). Blocking was performed with a 10% FBS solution (Gibco) in PBS 1 × (Sigma-Aldrich), incubating for 5 min at RT.

The coverslips were incubated with primary antibodies (1:400 dilution in 10% FBS/PBS 1 ×) against PLP (Abcam-Ab28486) and CNPase (Abcam-Ab6319), overnight at 4 °C, before being washed with PBS 1 × (Sigma-Aldrich). Then, samples were incubated with DAPI (Thermo Fisher Scientific) and secondary antibodies: anti-mouse (Alexa Fluor 488 goat anti-mouse—Thermo Fisher Scientific) and anti-rabbit (Alexa Fluor 488 goat anti-rabbit—Thermo Fisher Scientific) (both 1:400 in 10% FBS/PBS 1 ×), for 1 h in the dark at 37 °C. They were then rinsed three times with PBS 1 ×. For slide assembly, a drop of mounting medium (Dako) was added. The slides were dried at RT and sheltered from light, then read on a Cytation 5 (BioTek). The analyses were performed using ImageJ software (National Institutes of Health, NIH)^128^.

### RNA isolation and cDNA synthesis

MO3.13 cells were lysed with trizol and chloroform was used for phase separation. For RNA precipitation, isopropanol 100% was added, and the RNA pellet was washed three times in ice-cold 75% ethanol by centrifugation. Then, RNA was resuspended in nuclease-free water, and quantified using a DeNovix spectrophotometer. RNA quality was tested with 1% agarose gel electrophoresis, checking 18S/28S band integrity. Finally, reverse transcription was performed using a kit (GoScript Reverse Transcriptase Kit, Promega, Madison, WI, USA) according to manufactures’ instructions in a 20-µL reaction wit 1,6 µg of total RNA.

### Real-time quantitative polymerase chain reaction (RT-qPCR)

Quantitative PCR was performed on cDNA from MO3.13 cells diluted 1:10 using the qPCR Green Master Low Rox (Cellco) mastermix containing forward primer, reverse primer and diethyl pyrocarbonate (DEPC)-treated water. The RT-qPCR reaction was performed in a CFX384 Touch Real-Time PCR Detection System (Biorad). Cycling conditions were set as follows: after initial activation at 50 °C for 2 min and 40 cycles of 95 °C for 2 min, 95 °C for 15 s and 60 °C for 1 min, then melt curve analysis was performed by heating samples from 65 °C to 99 °C (1 °C increment changes at 5 s intervals), in order to evaluate primer specificity. All sample measurements were performed in duplicate.

Intron-spanning primers for RT-qPCR were designed with PrimerBlast, and used at 200 nM for PLP and CNP genes. Beta-actin (ACTB) was used as housekeeping gene (primer concentration: 100 nM). For primer sequences, see Supplementary Table S1. The efficiency of the primers used ranged from 95–100%. Data were normalized to the expression of ACTB gene and relative quantification value of each target gene was analyzed using a comparative CT method^129^.

## Supplementary information

Supplementary file 1

## Abbreviations

ACC: Anterior cingulate cortex
ACN: Acetonitrile
ACTB: Beta-actin
CNPase: 2′,3′-Cyclic-nucleotide-3-phosphodiesterase
COPS8: COP9 signalosome complex subunit 8
DIA: Data-independent acquisition
DLPFC: Dorsolateral prefrontal cortex
DMEM: Dulbecco’s modified eagle medium
DTT: Dithiothreitol
eIF2: Eukaryotic initiation factor -2
ESI: Electrospray ionization
FBS: Fetal bovine serum
FC: Fold change
HMG-CoA: 3-Hydroxy-3-methylglutaryl-CoA
hnRNP: Nuclear heterogeneous ribonucleoprotein
IAA: Iodoacetamide
IPA: Ingenuity pathway analysis
MBP: Myelin basic protein
MS: Mass spectrometry
NMDAR: N-methyl-d-aspartate receptor
NO: Nitric oxide
OFC: Orbitofrontal cortex
OPCs: Oligodendrocyte progenitor cells
PBS: Phosphate buffered saline
PDGFα: Alpha-type platelet-derived growth factor
PFA: Paraformaldehyde
PLP: Proteolipid protein
PSMD12: 26S proteasome non-ATPase regulatory subunit 12
RAD21: Double-strand-break repair protein rad21 homolog
RECQL: ATP-dependent DNA helicase Q1
RNF113A: Ring finger protein 113A
RT: Room temperature
SCP-2: Sterol carrier protein 2
T3: Triiodothyronine
TEAB: Tetraethylammonium bromide
TFA: Trifluoroacetic acid
TIM13: Inner mitochondrial membrane translocase 13
TOM34: Outer mitochondrial membrane translocase 34
TRIM28: Tripartite motif-containing 28
TRX: Thioredoxin
UBFD1: Ubiquitin family domain containing 1
