# Supplementary material for: Ubiquitin–proteasome system, lipid metabolism and DNA damage repair are triggered by antipsychotic medication in human oligodendrocytes: implications in schizophrenia
Source: Sci Rep. 2020 Jul 28;10:12655. doi: 10.1038/s41598-020-69543-5 (PMC7387551; doi:10.1038/s41598-020-69543-5)
Supplement: Supplementary file 1 [file 41598_2020_69543_MOESM1_ESM.pdf]

Ubiquitin-proteasome system, lipid metabolism and DNA damage repair are triggered by antipsychotic medication in human oligodendrocytes: implications in schizophrenia

Gabriela Seabra, Valéria de Almeida, Guilherme Reis-de-Oliveira, Fernanda Crunfli, André Saraiva Leão Marcelo Antunes[1], Daniel Martins-de-Souza[*]

Supplementary material

Supplementary table S1: Primers used for RT-qPCR (ACTB, PLP and CNPase forward and reverse sequences).

Primer	Function	Sequence
ACTB_Foward	Housekeeping	AGAGCTACGAGCTGCCTGAC
ACTB_Reverse	Housekeeping	AGCACTGTGTTGGCGTACAG
CNPase_Foward	Oligodendrocyte Marker	AGGACCTCTGGGCTTTGATTCCAT
CNPase_Reverse	Oligodendrocyte Marker	ACTCCAGCTTGGGAAATAGCAGGA
PLP1_Foward	Oligodendrocyte Marker	GCTGATGCCAGAATGTATGGTG
PLP1_Reverse	Oligodendrocyte Marker	CAATCATGAAGGTGAGCAGGG
